# Development of Novel Composite Biocompatible Materials by Surface Modification of Porous Inorganic Compounds Using Bambus[6]Uril

**DOI:** 10.3390/ma16237257

**Published:** 2023-11-21

**Authors:** Gulstan Zhumabayeva, Pana Turebayeva, Arthur Ukhov, Dmitry Fedorishin, Alexander Gubankov, Venera Luchsheva, Irina Kurzina, Abdigali Bakibaev, Roza Ryskaliyeva, Gulnara Abdullina, Saltanat Bolysbekova, Rakhmetulla Yerkassov

**Affiliations:** 1 Department of Chemistry, L.N. Gumilyov Eurasian National University, Astana 010000, Kazakhstan; gulistan2009@mail.ru (G.Z.); erkass@mail.ru (R.Y.); 2 Faculty of Chemistry, National Research Tomsk State University, 634028 Tomsk, Russia; artyryxov1@gmail.com (A.U.); strix187@yandex.ru (D.F.); 4.4.gub4nk0v@gmail.com (A.G.); kuscherbaeva_venera@mail.ru (V.L.); kurzina99@mail.ru (I.K.); bakibaev@mail.ru (A.B.); 3 Faculty of Chemistry and Chemical Technology, Department of General and Inorganic Chemistry, Al-Farabi Kazakh National University, Almaty 050000, Kazakhstan; roza12_11_64@mail.ru; 4 Faculty of Engineering, Department of Mechanics and Oil and Gas Engineering, Toraigyrov University, Pavlodar 140008, Kazakhstan; gulnara_1277@mail.ru; 5 Higher School of Scientific Research, Astana International University, Astana 010000, Kazakhstan; bolysbekova.s@mail.ru

**Keywords:** bambus[6]urils, hydroxyapatite, diatomite, biocompatible materials, hemolytic effect, plasma protein adsorption

## Abstract

In this present investigation, a novel series of composite materials based on porous inorganic compounds—hydroxyapatite and diatomite—have been innovatively formulated for the first time through surface modification employing the promising macromolecular compound, bambus[6]uril. The process entailed the application of a bambus[6]uril dispersion in water onto the surfaces of hydroxyapatite and diatomite. Extensive characterization was carried out, involving IR spectroscopy and SEM. The materials underwent assessment for hemolytic effects and plasma protein adsorption. The results revealed that materials containing surface-bound bambus[6]uril did not demonstrate inherent hemolytic effects, laying a robust groundwork for their use as biocompatible materials. These findings hold significant promise as an alternative pathway for the development of durable and efficient bio-composites, potentially unveiling supramolecular strategies incorporating encapsulated bambus[6]urils in analogous processes.

## 1. Introduction

In the contemporary era of personalized medicine, the necessity arises to develop implant materials that strike an optimal balance across various characteristics. These aspects encompass composition, shape, structure, mechanical properties, biocompatibility, and the potential to stimulate vessel or bone growth. In the context of implants or materials for wound treatment, all these attributes collectively determine the material’s ability to interact with the surrounding tissue. Bioactive materials can serve as a scaffold for the development of new tissue [1]. Conversely, a material interacting within the body’s internal environment should ideally exhibit minimal toxicity toward cells and tissues. This emphasizes the nature and extent of interaction between biomaterials and host tissues, presenting one of the critical challenges in biomaterial research [2,3,4]. Biocompatibility is defined by the immune response or inflammatory reactions of surrounding tissue systems in reaction to the presence of foreign entities within the body.

As clinical demands for all biomedical devices reach unprecedented heights, a meticulous surface modification process becomes imperative prior to their integration into the human body. Consequently, the development of materials that achieve a delicate equilibrium between biocompatibility and distinctive biological activity poses a significant challenge.

Moreover, contemporary clinical treatment modalities rely on the oral or intravenous administration of a medicinal agent, leading to a rapid surge in the drug’s concentration in the bloodstream immediately after administration. Following administration, the drug’s bloodstream concentration may reach toxic levels, only to subsequently fall below the therapeutic range, thereby compromising the efficacy of the therapy [5].

Implants designed for drug release have emerged as a promising alternative to conventional oral and intravenous drug delivery methods, presenting a variety of clinical treatment possibilities. Currently, established materials used for creating drug-releasing implants include titanium nanotubes, porous silicon, polymers, hydrogels, and microtechnologies. Drug-releasing implants offer the potential for sustained, remotely controlled, programmable, and localized drug release at specific sites, thereby enhancing therapeutic efficacy while minimizing adverse effects for patients. These capabilities extend beyond the scope of traditional systemic drug administration [6].

Modified hydroxyapatite (**HA**), enriched with bioactive compounds, serves as a tool for creating materials that have the potential to possess predetermined properties. Hydroxyapatite (**HA**) constitutes a fundamental building block of bones (accounting for around 50% of the total mass) and teeth (96% in enamel), and it is present in both synthetic and natural forms. Within the medical field, synthetic hydroxyapatite (**HA**) is used as a filler for the restoration of lost bone segments and as a coating on implants to stimulate the growth of new bone [7].

Another notable material of interest is diatomite (**DA**), the fossilized residue of diatom planktonic algae found in aquatic environments worldwide. Mainly composed almost entirely of silicon dioxide (SiO_2_) [8], diatomite (**DA**) is non-toxic, odorless, abundantly available in nature, easily purified, and relatively cost-effective. Among natural materials, diatomite (**DA**) boasts unique properties, including high porosity (10–100 nm), permeability, fine particle size, substantial surface area (29 m^2^/g), and pore volume (0.09 cm³/g) [9]. It also exhibits low thermal conductivity and chemical inertness [10], making it suitable for applications in various domains such as construction, water filtration, agriculture, and more [11]. Diatomite (**DA**) finds use as a contact insecticide in arid climates as well as a soil conditioner and additive in animal feed and human food products [11].

Presently, surface modification techniques for porous materials through the impregnation of biologically active compounds, including macromolecular entities [12], are gaining prominence. These approaches enable precise control over the release of antibiotics, pharmaceutical agents, bioactive substances, and cells [13]. Macromolecular compounds are often preferred over other drug delivery systems, such as dendrimers, liposomes, micelles, carbon nanotubes, hydrogels, and polymers, due to their stability and controlled drug release rates [14,15,16,17,18].

In the context of surface modification for porous materials, macromolecular systems based on bambus[6]uril are particularly suitable. These macrocyclic compounds, interconnected through bridges along the equator of the macrocycle, consist of dimethylglycoluril units [19]. Notably, these macrocyclic entities possess the unique ability to encapsulate therapeutic agents, thus facilitating controlled and sustained release under various influences, including light exposure, pH variations, and temperature fluctuations [20]. Bambus[6]uril (**Bu[6]**) engages in weak hydrogen bonding with anions within its hydrophobic cavity [21]. Its more positively charged electrostatic region attracts anions, while the portal carbonyl oxygen atoms create a negative region that can interact with positively charged particles. Materials based on the synergy of methylviologen and **Bu[6]** have been utilized in the development of energy storage systems and the engineering of light-emitting diodes [22]. **Bu[6]** also serves as a versatile carrier in liquid membranes for applications like electromembrane extraction [23].

Therefore, surface modification techniques of inorganic porous materials using macrocyclic compounds, including **Bu[6]**, hold significant promise for practical applications across various fields. This potential is further highlighted by the limited instances of such techniques documented in the literature, with only one example based on titanium nickelide [17]. In this research, **Bu[6]** has been applied to the surface of porous materials (hydroxyapatite and diatomite) for the first time, employing various techniques. This advancement lays the groundwork for loading the **Bu[6]** cavity with therapeutic agents, enhancing the porous materials’ therapeutic potential, and expediting osteogenesis. Hence, the objective of this study is to assess how **Bu[6]** deposition methods influence the continuity, structure, and in vitro hemocompatibility of the porous surfaces of hydroxyapatite and diatomite.

## 2. Materials and Methods

Glyoxal was purchased from Novochem (Tomsk, Russia) and silver nitrate from Reachim (Sverdlovsk, Russia). All other chemicals were from Merck/Sigma-Aldrich (Darmstadt, Germany).

### 2.1. Synthesis of **HA**

Hydroxyapatite (**HA**) synthesis was accomplished through a liquid-phase method employing microwave irradiation at a pH of approximately 11, following the provided scheme [24]:

10Ca(NO_3_)_2_ + 6(NH_4_)_2_HPO_4_ + 8NH_4_OH → Ca_10_(PO_4_)_6_(OH)_2_ + 20NH_4_NO_3_ + 6H_2_O

For the preparation of initial solutions, the following reagents were used: calcium nitrate tetrahydrate Ca(NO_3_)_2_ × 4H_2_O, ammonium hydrogen phosphate (NH_4_)_2_HPO_4_, 25% aqueous ammonia solution, and distilled water.

A 300 mL chemical beaker was utilized to create a solution with a concentration of 0.5 mol/L, where Ca(NO_3_)_2_ × 4H_2_O (47.20 g) was dissolved in 200 mL of distilled water. Similarly, (NH_4_)_2_HPO_4_ (15.84 g) was dissolved in 200 mL of distilled water to form a solution with a concentration of 0.3 mol/L. The prepared solutions were combined in a single 500 mL beaker and adjusted to pH 11 by the gradual addition of a 25% aqueous ammonia solution under continuous stirring. The beaker with the reaction mixture was covered with a film, placed in a microwave oven, and set to a working power of 100–150 W, thereby initiating the heating process. Microwave irradiation continued until the reaction mixture reached its boiling point. To prevent local overheating, the contents of the reaction vessel were periodically stirred at precise intervals of 15 min during the microwave synthesis. The total duration of microwave heating was 45 min. Subsequently, the beaker with its contents was taken out and left at room temperature for 48 h to facilitate the formation of the hydroxyapatite phase. The resulting precipitated **HA** was filtered, dried in a drying oven at 110 °C until a constant mass was achieved (approximately 20 h), and then milled to obtain a uniform state. From the resulting hydroxyapatite **HA** powder, carriers were shaped into tablets, 2 cm in width and approximately 1 mm in thickness, and subjected to calcination at 600 °C.

### 2.2. **Bu[6]** Synthesis

**Bu[6]** was synthesized, isolated, and purified using a traditional method [19] based on the preliminary formation of 2,4-dimethylglycoluril, followed by acid-catalyzed cyclization with formaldehyde to produce the desired product.

**Figure 1:**
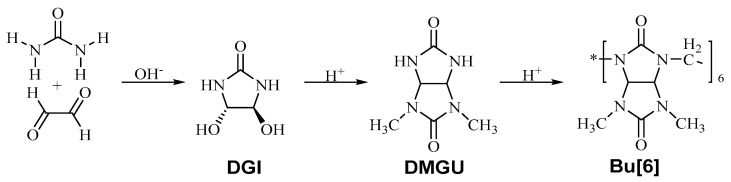
Visual Representation of the **Bu[6]** Application onto **HA** and **DA** Surfaces.

In the initial stage, the synthesis of 4,5-dihydroxyimidazolidinone-2 (**DGI**) was conducted (Figure 1). The synthesis took place in a round-bottom flask equipped with a reflux condenser and a magnetic stirrer. Urea (50 g) was loaded into the flask, and 100 mL of a 40% glyoxal solution was added. The pH was adjusted to 7 by the addition of a 10% solution of sodium hydroxide. The synthesis was carried out for 7 h with continuous stirring at a temperature of 50 °C. Upon completion of the reaction and subsequent cooling of the solution to room temperature, the pH was increased to 9 using a 10% solution of sodium hydroxide. The solution was refrigerated for three days to facilitate the crystallization of 4,5-dihydroxyimidazolidinone-2. The resulting crystals were filtered and air-dried, providing 61.8 g (60.5%) of **DGI** with a melting point of 160–162 °C. The synthesized product exhibited a white crystalline appearance.

Afterward, the acquired **DGI** was utilized for the synthesis of 2,4-N-dimethylglycoluril (**DMGU**). The synthesis process took place in a round-bottom flask furnished with a reflux condenser and magnetic stirrer. The flask contained 40 g of 4,5-dihydroxyimidazolidinone-2 and 35.2 g of dimethylurea, along with 100 mL of water. The pH was adjusted to 2 using concentrated hydrochloric acid. The synthesis lasted for 4 h at 90 °C. Upon completion, the solution was partially evaporated, and 10 mL of acetone was added. The resulting mixture was refrigerated for 48 h, leading to the formation of precipitated sediment. The resulting precipitate underwent two rounds of recrystallization from acetone, yielding 31.2 g (48%) of **DMGU** with a melting point of 254–256 °C. The measurement was conducted within the temperature range of room temperature to 350 °C, with an increment rate of 5 °C per minute. The synthesized product manifested as pure white crystals.

1H NMR (DMSO-d6, δ, ppm): 2.64 (6H, s, CH3), 5.12 (2H, s, CH), 7.54 (2H, s, NH).

13C NMR (CDCl3, δ, ppm): 158.22 and 160.20 (C=O), 28.22 (CH3), 76.67 (C-H).

The final phase entailed the synthesis of **Bu[6]**. This process occurred in a chemical flask equipped with a magnetic stirrer. The flask contained 30 g of **DMGU**, 30 mL of concentrated hydrochloric acid, and 45 mL of a 40% formaldehyde solution. The resulting mixture was continuously stirred for 24 h. Subsequently, the mixture was poured into 400 mL of water and agitated for 2 h. The suspension was then filtered, and the precipitate was air-dried, yielding 5.2 g (16%) of bambus[6]uril. The product underwent recrystallization with concentrated hydrochloric acid.

The obtained **Bu[6]** was identified using NMR and IR spectroscopic techniques. The IR spectrum displayed peaks at 2940 cm^−1^ (CH_2_), 1681 cm^−1^ (C=O), 1446 cm^−1^ (CH_3_), 789 cm^−1^, and 656 cm^−1^ (C-H).

The NMR spectrum of **Bu[6]** showed chemical shifts in the ^1^H NMR (DMSO-d_6_/CDCl_3_ (1:1), TMS) as follows: 5.29 (s, 12H, CH), 5.06 (s, 12H, CH_2_), and 2.51 (s, 36H, CH_3_). The ^13^C NMR (DMSO-d_6_/CDCl_3_ (1:1), TMS) exhibited shifts at: 159.32 (C=O, Me_2_Urea), 158.45 (C=O, Urea), 67.82 (C-H, CH_3_), 48.78 (CH, –CH_2_–), and 31.06 (CH, CH_3_-).

### 2.3. Refining С**DA** for Enhanced Purity

For research purposes, diatomaceous earth (**DA**) obtained from “Quant” LLC (Penza Region) was utilized in different forms—in its original state (**IDA**) and further purified (**CDA**)—to remove various cations and anions. The purification of diatomaceous earth was carried out using a boiling method in an 18% hydrochloric acid solution [25]. To accomplish this, a sample of diatomaceous earth (m = 40 g) was placed into a solution of 18% hydrochloric acid (V = 300 mL). Subsequently, the solution was brought to a boil and left to stand for 24 h. Following this, the diatomaceous earth was filtered and air-dried at room temperature until a constant mass was achieved. Both the untreated **IDA** and the refined **CDA** were compressed into tablet-shaped carriers, each measuring 2 cm in width and approximately 1 mm in thickness, and then subjected to calcination at 600 °C.

### 2.4. Physicochemical Characterization of Composite Materials

#### 2.4.1. FTIR

The identification and structural analysis of the **Bu[6]** samples were performed using Fourier-transform infrared (FT-IR) spectroscopy with a Nicolet 6700 infrared spectrometer from Thermo Fisher Scientific. The samples were examined using the attenuated total reflection (ATR) method over the spectral range of 400 to 4000 cm^−1^, with a resolution of 4 cm^−1^. The resulting reflection spectra were transformed into absorption spectra using the Kubelka-Munk transformation.

#### 2.4.2. NMR Spectroscopy

NMR spectroscopy of **Bu[6]** and **DMGU** was conducted using a Bruker Avance 400 III HD NMR spectrometer in DMSO-d6 solution at a temperature of 25 °C. The proton nuclei were operating at a frequency of 400 MHz, while the carbon nuclei were at 100 MHz. Samples for NMR analysis were prepared at a concentration of 10 mg/mL with a volume of 0.5 mL.

#### 2.4.3. Scanning Electron Microscopy (SEM)

The analysis was performed using a QUANTA 200 3D system (EDAX, Tilburg, The Netherlands) equipped with both electron and focused-beam capabilities. The system operated with a continuous range of accelerating voltages from 200 to 30000 V. In ESEM mode, a remarkable resolution of 3.5 nm was achieved at 30 kV, while in low vacuum mode at 3 kV, a resolution of <15 nm was attained.

#### 2.4.4. X-ray Diffraction (XRD)

The investigation of crystalline powders of **HA** and **Bu[6]** was conducted using X-ray phase analysis. The sample analysis was carried out on a XRD-7000 X-ray diffractometer (Shimadzu, Japan) with a Cu anode, an X-ray wavelength of Kα(Cu) = 1.5406 Å, a measurement range of 5–50° in 2θ, and a measurement speed of 30°/min. Identification of the analyzed samples was achieved by matching their spectra with the diffraction patterns of reference substances using diffraction data from the Cambridge Crystallographic Data Centre database.

**Figure 2:**
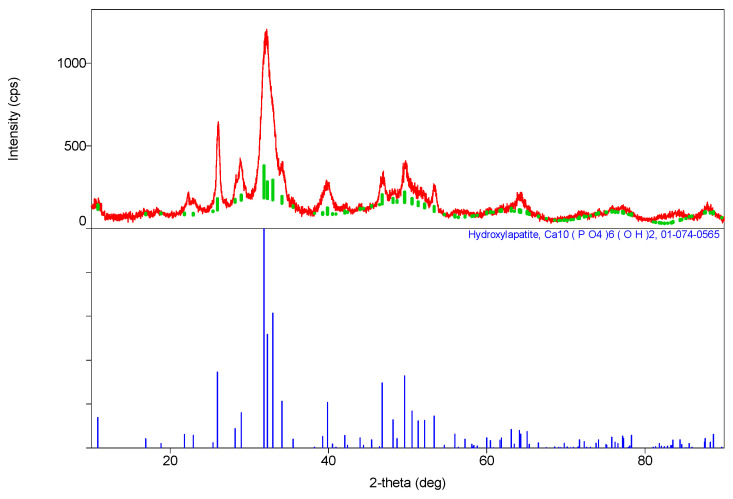
Diffractogram of synthesized stoichiometric **HA** (blue line stands for standard data and red line stands for experimental data).

The XRD findings demonstrate (Figure 2) the establishment of a hexagonal crystal system during the synthesis of stoichiometric **HA**, gr. P63\m, with the overall formula described as Ca_10_(PO_4_)_6_(OH)_2_ (Table 1). The unit cell parameters of the synthesized **HA** closely correspond to the data in the reference table (JCPDS data, №9-432, [26]).

The melting points of the samples were determined using the BÜCHI Melting Point M-560 (BÜCHI, Landswill, Bern, Switzerland) apparatus in an open capillary setup.

### 2.5. Assessment of Hemocompatibility of Biocomposites

One method to evaluate the overall cytotoxicity of compounds and materials is to study their hemolytic activity [27,28]. When the material exhibits poor hemocompatibility, the destruction of erythrocytes leads to the release of hemoglobin into the solution. Increased hemoglobin presence in the solution results in higher optical density. Therefore, elevated optical density indicates lower hemocompatibility of the material.

Peripheral blood samples were collected from consenting healthy volunteers.

To assess the hemocompatibility of the examined samples, whole anticoagulated blood from a healthy donor was employed. The blood underwent centrifugation, leading to the separation of the erythrocyte mass. The resulting erythrocyte mass was then diluted with a sterile 1X PBS solution at 37 °C in a 1:9 ratio. The samples were placed in a standard 12-well cell culture plate and covered with the prepared blood-PBS solution at a ratio of 1 mL solution per 1 cm² of the sample’s surface area. Deionized water served as the positive control (100% hemolysis), while a 1X PBS solution acted as the negative control (0% hemolysis). Similarly, **IDA** and **CDA** samples were utilized as control materials. Subsequently, the plate was incubated in a thermostat at 37 °C for 60 min. After the incubation period, blood from the wells was transferred to centrifuge tubes and centrifuged for 5 min at 3000 rpm to separate the remaining erythrocytes. The supernatant was carefully removed and transferred to a standard 96-well plate for spectroscopic analysis. Optical density measurements were performed using the state-of-the-art IFA-reader Tecan Infinite F50 (Tecan Inc., Morrisville, NC, USA) operating at a wavelength of 492 nm.

The percentage of hemolysis was determined as the average of three replicates and calculated using the formula [27]:

$$\mathrm{Hemolisys},\;\%=\frac{\left(OD_{\mathrm{test}\;\mathrm{sample}}-OD_{\mathrm{control}}^{\mathrm{negative}}\right)}{\left(OD_{\mathrm{control}}^{\mathrm{positive}}-OD_{\mathrm{control}}^{\mathrm{negative}}\right)}\times 100\%$$

The Ethical Committee of Tomsk State University approved the study design and the recruitment of subjects. Subjects provided written, informed consent. The relevant guidelines and regulations were followed when performing the experiments.

### 2.6. Evaluation of Plasma Protein Adsorption by Biocomposites

The adsorption of plasma proteins onto our developed samples was investigated using a modified solution depletion method. This approach involves two quantitative determinations of protein concentration in the blood plasma—before and after incubation of the samples [28].

Peripheral blood was obtained from healthy volunteers after receiving their written consent.

Plasma was separated from whole anticoagulated blood obtained from a healthy donor through centrifugation. The protein content within the plasma was determined utilizing the biuret reaction method. Subsequently, 2 mL of plasma was applied to the samples and incubated at 37 °C for 24 h. Following incubation, another assessment of protein concentration was conducted. The variance in protein concentration between the intact plasma and the post-incubation sample indicated the extent of protein adsorption by the samples. A greater disparity between these measurements indicated a higher degree of protein adsorption on the sample.

Under alkaline conditions, serum proteins interacted with copper sulfate, resulting in the formation of complex compounds exhibiting a violet color due to the presence of peptide bonds. The intensity of this color was directly proportional to the protein concentration in the solution.

During the experiment, 2 mL of plasma was applied to the samples and incubated at 37 °C for 24 h. After the incubation period and removal of the samples from the solution, a biuret reaction was conducted, followed by an optical density measurement.

The biuret reaction involved the mixing of 0.1 mL of blood plasma with 5.0 mL of a working solution of the biuret reagent, with precautions taken to prevent the formation of foam. A concurrent control experiment was conducted in parallel, involving the mixing of 0.1 mL of a 0.9% sodium chloride solution with 5 mL of the prepared working biuret reagent solution. After a precise interval of 30 min (but no later than one hour), the optical density of the solution was recorded using the Tecan Infinite F50 microplate reader (Tecan Inc., Morrisville, NC, USA) at a defined wavelength of 492 nm, with reference to the control.

## 3. Results and Discussion

In this study, we examined the influence of the macromolecular compound—bambus[6]uril (**Bu[6]**)—on the biocompatibility of hydroxyapatite (**HA**) and diatomite (**IDA** and **CDA**) to establish a matrix for prospective biomedical composite materials.

**Figure 3:**
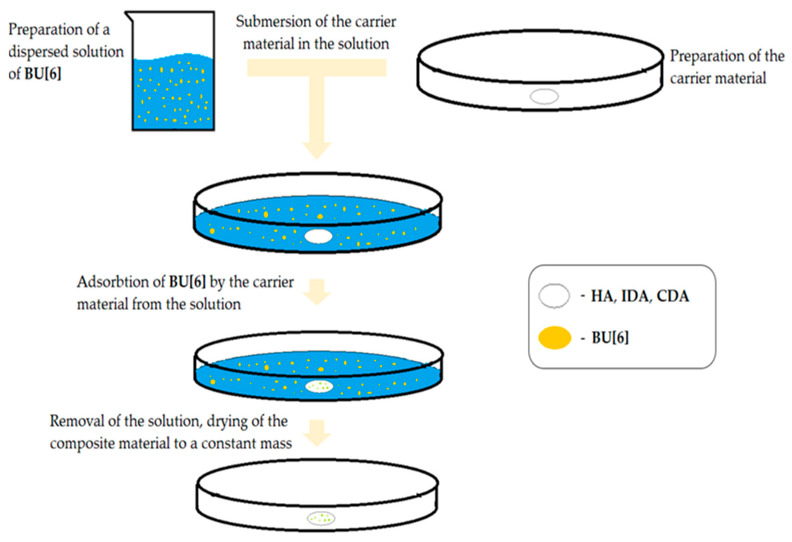
Illustration of the Deposition Method of **Bu[6]** onto **HA** and **DA**.

To apply **Bu[6]** onto porous inorganic surfaces, an immersion method was employed (Figure 3). For the execution of this methodological approach, a dispersion of **Bu[6]** was prepared in water at a concentration of 1 mg/mL in deionized water. The volume of solution was 15 mL. Subsequently, the **HA**, **IDA**, and **CDA** carriers were subjected to immersion in the **Bu[6]** solution and left to interact for a duration of 40 min. Post-immersion, the solution was decanted, and the resulting composite specimens (**HA + Bu[6]**, **IDA + Bu[6]**, and **CDA + Bu[6]**) were subjected to gentle air-drying at room temperature until a consistent weight was achieved.

The ensuing composite materials underwent comprehensive analysis through IR spectroscopy and SEM. The quantification of applied **Bu[6]** was determined via gravimetric assessment. The residual solution, after the application process, was dried to a steady state, and the remaining **Bu[6]** content was precisely measured. The quantity of **Bu[6]** applied was 10 mg.

**Figure 4:**
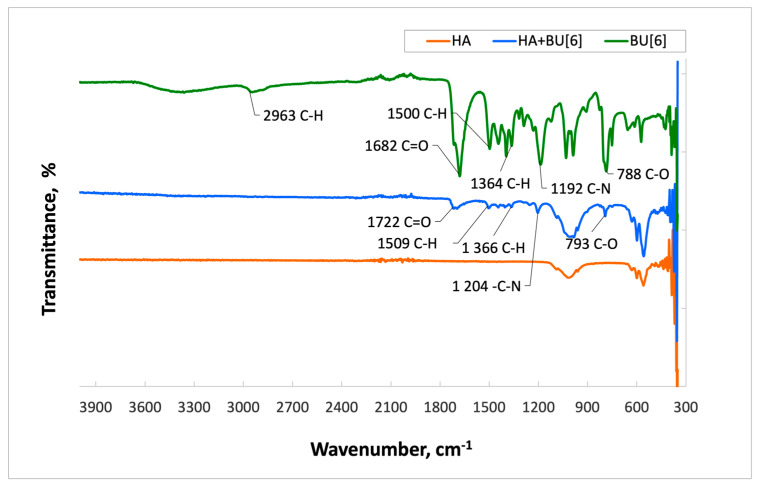
Infrared Spectra of Samples: **Bu[6], HA + BU[6], HA**.

The spectrum of **HA** with **Bu[6]** (Figure 4) displays characteristic absorption bands of **Bu[6]** at 1716 cm^−1^ and 1696 cm^−1^, which correspond to the valence vibrations of carbonyl (C=O) groups within the glycoluril units. The presence of two distinct signals suggests the existence of non-equivalent carbonyl groups in **Bu[6]**, namely Me_2_Urea (a fragment of dimethylurea) and Urea (a fragment of urea). In the **HA + Bu[6]** spectrum, a shift of these characteristic absorption bands of the C=O functional groups by 19 cm^−1^ towards the short-wavelength region compared to the IR spectrum of **Bu[6]** is observed, indicating an interaction with the **HA** surface through the C=O gateway groups of bambusuril. Additionally, the spectrum exhibits an absorption band at 1502 cm^−1^, corresponding to the CH_3_ group, with C-H bonds identified at 792 cm^−1^. Deformation vibrations of CH_2_ groups are identified in the range of 1200–1500 cm^−1^.

**Figure 5:**
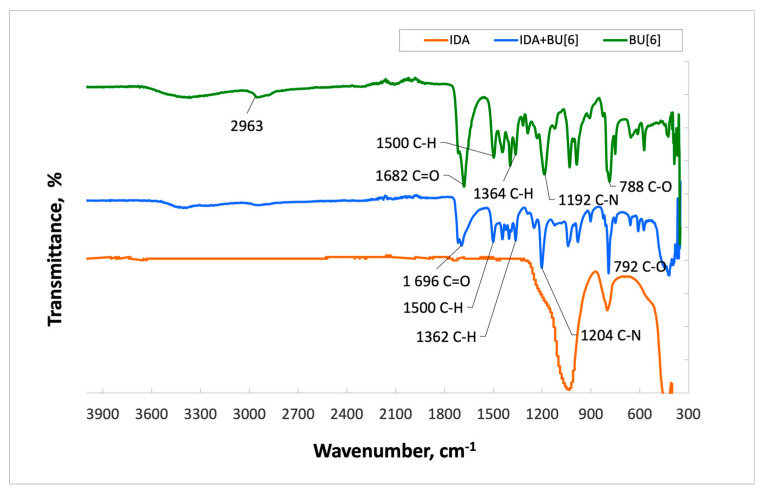
Infrared Spectra of Samples: **Bu[6]**, **IDA** + **Bu[6]**, **IDA**.

In the **IDA + Bu[6]** spectrum (Figure 5), characteristic absorption bands of **Bu[6]** are observed at 1716 cm^−1^ and 1694 cm^−1^, corresponding to the carbonyl (C=O) stretching vibrations in the N,N-2,8-dimethylglycoluril units. Similar to the **HA + Bu[6]** spectra, a shift of carbonyl groups is evident, with shifts of 19 cm^−1^ and 16 cm^−1^, respectively, compared to the original **Bu[6]** spectrum. The peak at 1500 cm^−1^ is attributed to the CH_3_ group, and C-H bonds are also identified at 792 cm^−1^. Deformation vibrations of CH_2_ groups are identified within the range of 1200–1500 cm^−1^. The absorption band at 2941 cm^−1^ corresponds to the carbonyl stretching vibrations in the methylene bridges.

**Figure 6:**
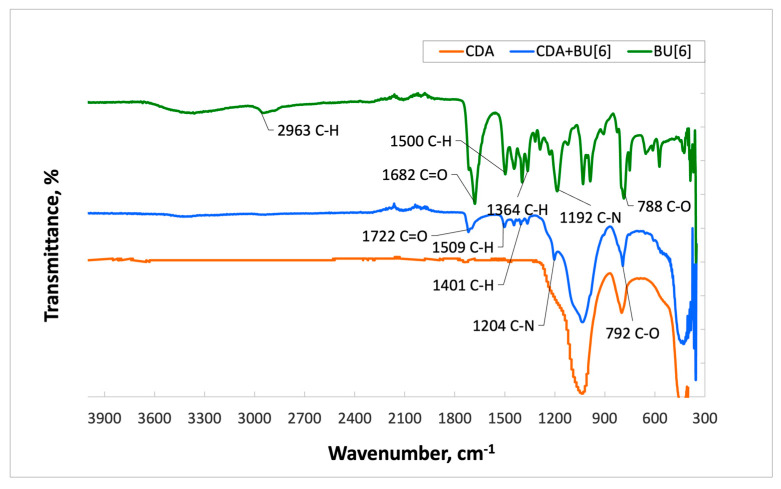
Infrared Spectra of Samples: **Bu[6]**, **CDA + Bu[6]**, **CDA**.

Upon examination of the IR spectrum of **CDA**, a similar pattern is observed (Figure 6). The obtained results indicate the presence of **Bu[6]** on the surfaces of both **IDA** and **CDA**.

**Figure 7:**
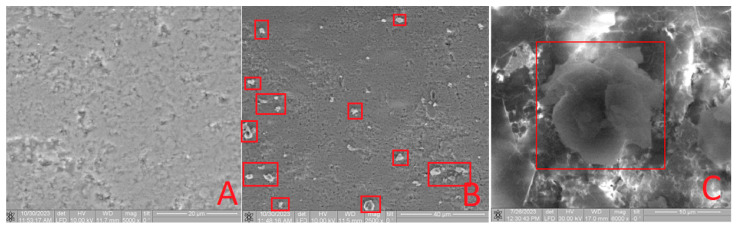
SEM Image of the **HA** (**A**) and **Bu[6] + HA** samples with varying resolution (**B**,**C**), highlighting the conglomerate of **Bu[6]** molecule ensembles in a red square.

Furthermore, the characterization of the obtained composites included a thorough examination using SEM. In Figure 7A, the surface of **HA** before modification is depicted. Following the **HA** modification, an uneven distribution of molecules across the surface is evident. Notably, the SEM image of the **Bu[6] + HA** sample (Figure 7B,C) vividly reveals a conglomerate ensemble of **Bu[6]** molecules, self-assembled into structures approximately 10 µm in size. This observation strongly confirms the presence of **Bu[6]** on the surface of the composite material **Bu[6] + HA**.

**Figure 8:**
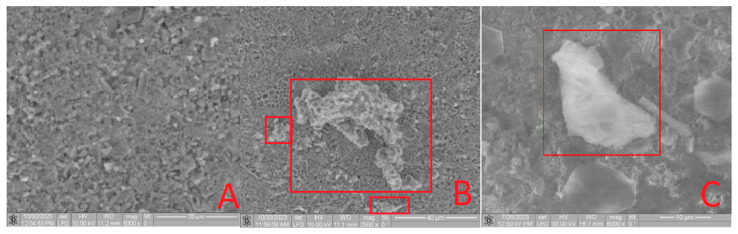
SEM image of the **IDA** (**A**) and **Bu[6] + IDA** sample with varying resolution (**B**,**C**), highlighting the conglomerate of **Bu[6]** molecule ensembles in a red square.

Upon SEM analysis of the **Bu[6] + IDA** sample (Figure 8B,C), a comparable phenomenon is apparent, revealing a conglomerate assembly of **Bu[6]** molecules on the **IDA** surface with dimensions spanning 5 to 15 µm. The unexpected formation of these associates, contrary to the typical behavior of bambus[6]uril with a molecule diameter of ≤10 Å [4], can be attributed to the inherent tendency of the dispersed **Bu[6]** solution in water to engage in such associative interactions.

**Figure 9:**
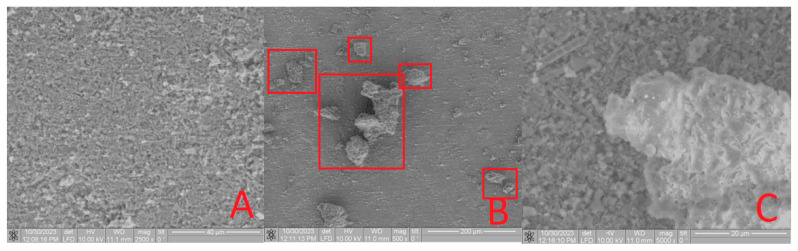
SEM image of the **CDA** (**A**) and **Bu[6] + CDA** samples with varying resolution (**B**,**C**), highlighting the conglomerate of **Bu[6]** molecule ensembles in a red square.

The **CDA** surface (Figure 9A) and the surfaces of the **Bu[6]** **+ CDA** material exhibit comparable results. The larger diameter of the **Bu[6]** particles surpasses both the pore size of the diatomite carriers (**IDA, CDA**) and the hydroxyapatite (**HA**), indicating their pore overlap and the surface retention of the **Bu[6]** macrocycle. The collective evidence corroborates the presence of **Bu[6]** on the surfaces of **Bu[6] + HA, Bu[6] + IDA,** and **Bu[6] + CDA**.

The toxicological effects of the developed biocomposites were assessed through the investigation of their hemocompatibility and plasma protein adsorption.

One approach to evaluating the overall cytotoxicity of a material involves examining its hemolytic activity. The hemolysis test is based on the degree of erythrolysis and hemoglobin dissociation upon material contact with erythrocytes in vitro.

Hemocompatibility and thromboresistance of biomaterials constitute crucial components of their biocompatibility. When foreign materials come into contact with blood, coagulation or thrombus formation may occur. Thromboresistance, the ability of a biomaterial to prevent thrombus formation, is a pivotal characteristic of biological compatibility. Hemocompatibility, on the other hand, represents an aspect of material-blood interaction and can be viewed from various perspectives, influenced by both chemical and physical material properties [29].

The results of the experimental assessment of hemolytic activity levels of the **HA + Bu[6]** group samples are presented in Table 2, compared with individual hydroxyapatite **HA** and **bambus[6]uril Bu[6]**.

The examination of Table 2 indicates that the unaltered hydroxyapatite (**HA**) sample showed signs of hemolytic activity. Similar hemolytic tendencies were also observed in the **Bu[6]** and **HA + Bu[6]** samples. However, no statistically significant differences in the level of hemolysis between these samples and the negative control (**CTRL** 0%) were found (*p* > 0.05).

The hemolytic activity of the unmodified hydroxyapatite (**HA**) could potentially be attributed to the decrease in porosity caused by the treatment with various substances. According to existing literature, hemolysis on the surface of inert biomaterials is closely associated with the adsorption of plasma proteins, particularly fibrinogen, onto the material’s surface in contact with blood, where increased plasma protein adsorption corresponds to heightened hemolysis [19].

Hydroxyapatite is a porous material with small pore sizes (14–20 nm) [20], significantly limiting the potential for extensive plasma protein adsorption on its surface [30]. This limitation on adsorption may contribute to the noticeable hemolytic activity observed in the unmodified hydroxyapatite **HA** samples.

The research conducted demonstrated that the application of **Bu[6]** reduced the hemolytic activity of hydroxyapatite (**HA**), as observed in the **HA + Bu[6]** composite sample. However, statistically significant differences in the level of hemolysis between the **HA + Bu[6]** sample and the negative control (**CTRL** 0%) were not established (*p* > 0.05). According to existing literature, protein adsorption occurs on any abiotic surface, and the nature of the adsorbed protein layer depends on the magnitude and potential difference on the surface. If the material’s positive potential exceeds that of blood, the likelihood of thrombosis increases [31].

Based on the above, it can be speculated that **Bu[6]**, acting as a potent complexing agent, binds ions from the blood solution, thereby reducing the positive potential of the porous material’s surface, ultimately reducing the adhesion of blood cellular components and enhancing hemocompatibility.

To determine whether the level of hemolytic activity correlates with the degree of plasma protein adsorption on the samples, we examined the extent of the reduction in plasma protein concentration after incubation with the samples.

The data presented in Table 3 indicates that the **HA** group samples experienced a decrease in plasma protein concentration after incubation, which was statistically confirmed (*p* < 0.05). Moreover, the **HA** samples displayed higher protein adsorption compared to the modified **bambus[6]uril HA + Bu[6]** samples.

The research conducted suggests that the modification of hydroxyapatite (**HA**) samples with bambus[6]uril (**Bu[6]**) enhances their hemocompatibility and reduces adsorption.

In the subsequent phase of our study, we examined the biocompatible properties of intact diatomite (**IDA**), purified **CDA** diatomite, bambus[6]uril (**Bu[6]**), and their compositions (**IDA + Bu[6]** and **CDA + Bu[6]**).

According to the data presented in Table 3, the samples in the **Bu[6]** and **CDA + Bu[6]** groups exhibited some hemolytic activity; however, no statistically significant differences between these samples and the negative control were observed (*p* > 0.05). Conversely, samples from the other groups did not display any hemolytic activity (Table 4). The absence of hemolytic activity in these groups can be attributed to the inherently high biocompatibility of diatomite, as supported by existing literature [11].

The primary challenge of preventing unwanted blood coagulation upon contact with implanted materials and devices remains a persisting concern. This challenge stems from the absence of protective mechanisms present in healthy vascular endothelium that counteract thrombosis. Foreign materials, on the other hand, tend to promote blood clotting through a series of interconnected processes, including protein adsorption, platelet and leukocyte adherence, thrombin generation, and complement activation [19]. Given this context, the development of strategies that mitigate hemolysis while modifying biocompatible materials holds paramount significance.

It is important to note, however, that the level of hemolysis for biomaterials interacting with the body’s internal environment should not exceed 5% [27]. According to the experimental data, none of the samples surpassed this threshold, leading to the conclusion that all the tested samples demonstrate hemocompatibility.

As illustrated in Table 5, the unmodified **Bu[6]** samples (**IDA** and **CDA**) exhibited a decrease in plasma protein concentration after incubation, a phenomenon that was statistically confirmed (*p* < 0.05). These samples demonstrated higher adsorption compared to their modified counterparts with bambus[6]uril. Similarly, the **Bu[6]**-modified samples (**IDA + Bu[6]** and **CDA + Bu[6]**) also showed a decrease in protein concentration, but no statistically significant differences were observed between these samples and the negative control (*p* > 0.05).

The data obtained from Table 5 indicates that the modification of **Bu[6]** samples results in decreased protein adsorption, consequently enhancing their hemocompatibility.

It is crucial to emphasize that the hemolysis level of biomaterials within the body’s internal environment should not surpass 5% [27]. According to the findings of the experiments, none of the modified **Bu[6]** samples of hydroxyapatite and diatomite exceeded this threshold, leading to the conclusion that all the investigated samples exhibit hemocompatibility.

The reduction in hemolysis level and adsorption during the modification of **Bu[6]** samples is attributed to alterations in the material’s surface properties, including surface tension, free surface energy, roughness, and hydrophilicity. The deposition of **Bu[6]** onto the surfaces of porous materials induces changes in their surface charge, making them more akin to blood. Consequently, plasma protein adsorption diminishes, leading to reduced thrombogenicity and enhanced hemocompatibility of the developed biocomposites.

## 4. Conclusions

This study has pioneered the successful development of novel biocomposites by integrating bambus[6]uril onto various porous substrates, namely hydroxyapatite and **diatomite**. The composite materials, **Bu[6]** **+ HA**, **Bu[6]** **+ IDA**, and **Bu[6]** **+ CDA**, underwent comprehensive characterization using sophisticated techniques, including infrared spectroscopy (IR) and scanning electron microscopy (SEM). The investigation focused on examining the hemolytic effects and plasma protein adsorption of the materials. Notably, the findings revealed the formation of a complex assembly of **Bu[6]** molecules within the biocomposites, leading to a significant decrease in plasma protein adsorption and a substantial improvement in hemocompatibility.

**HA**, **IDA**, and **CDA** are well-known porous materials widely recognized for their strong biocompatibility. Consequently, the incorporation of bambus[6]uril onto the surfaces of these porous materials is expected to facilitate the creation of potential materials featuring an optimal microenvironment for osteogenesis and the controlled release of bioactive compounds, including antibacterial drugs. These innovative biocomposites exhibit promising potential for finely regulating biological responses through host-guest interactions enabled by **Bu[6]** immobilization on customized carriers, which will be the focal point of our upcoming research endeavors.

## Figures and Tables

**Table 1:** Phase composition of **HA** synthesis products.

Sample	The Inorganic Phase	Parameters of the Electronic Cell, Ǻ	
		a	c
Synthesis product (**HA**)	Ca_10_(PO_4_)_6_(OH)_2_	9411	6863
JCPDS data, №9-432	Ca_10_(PO_4_)_6_(OH)_2_	9418	6884

**Table 2:** Hemocompatibility level of **HA**, **Bu[6]**, and **HA + Bu[6]** group samples.

№	Sample	Hemolysis (%)
1	**HA**	1.6814 ± 0.0012 *
2	**Bu[6]**	0.2989 ± 0.0017
3	**HA + Bu[6]**	0.9884 ± 0.0033
4	CTRL 100%	100
5	CTRL 0%	0

Note: **HA**—hydroxyapatite; **Bu[6]**—bambus[6]uril; **CTRL**—control. *—hemolysis level significantly differs from positive control (*p* < 0.05).

**Table 3:** Plasma protein adsorption levels of **HA, Bu[6],** and **HA + Bu[6]** group samples.

№	Sample	Optical Density	Δ of Optical Density
1	**HA**	0.1359 ± 0.0115	0.1255
2	**Bu[6]**	0.2318 ± 0.0237	0.0382
3	**HA + Bu[6]**	0.2100 ± 0.0330	0.0600
4	CTRL (PBS)	0.1228 ± 0.0059	0.1472
5	CTRL (Plasma)	0.2700 ± 0.0164	0.0000

Note: **HA**—hydroxyapatite; **Bu[6]**—bambus[6]uril; **CTRL** (PBS)—empty experiment; **CTRL** (Plasma)—protein content in intact plasma.

**Table 4:** Hemocompatibility Levels of **IDA**, **CDA**, and **DA + Bu[6]** group samples.

№	Sample	Hemolysis (%)
1	**IDA**	0
2	**CDA**	0
3	**Bu[6]**	0.2989 ± 0.0017
4	**IDA + Bu[6]**	0
5	**CDA + Bu[6]**	0.7971 ± 0.0008
6	CTRL 100%	100
7	CTRL 0%	0

Note: **IDA**—intact diatomite; **CDA**—purified diatomite; **Bu[6]**—bambus[6]uril; **CTRL**—control.

**Table 5:** Plasma Protein Adsorption Levels of **IDA**, **CDA**, and **DA** + **Bu[6]** group samples.

№	Sample	Optical Density	Δ of Optical Density
	**IDA**	0.1386 ± 0.0133	0.1227
	**CDA**	0.1366 ± 0.0053	0.1247
1	**Bu[6]**	0.2318 ± 0.0237	0.0382
2	**IDA + Bu[6]**	0.1865 ± 0.0318	0.0835
3	**CDA + Bu[6]**	0.1946 ± 0.0084	0.0754
4	CTRL (PBS)	0.1228 ± 0.0059	0.1472
5	CTRL (Plasma)	0.2700 ± 0.0164	0.0000

Note: **IDA**—Intact Diatomite; **CDA**—Cleaned Diatomite; **Bu[6]**—Bambus[6]uril; **CTRL** (PBS)—Blank Experiment; **CTRL** (Plasma)—Protein Content in Intact Plasma.

## Data Availability

The data presented in this study are available in this article.
